# General Factors and Dental-Related Risk Factors for Postoperative Pneumonia or Infectious Complications: A Retrospective Study

**DOI:** 10.3390/jcm12103529

**Published:** 2023-05-17

**Authors:** Emiko Tanaka Isomura, Yukari Fujimoto, Makoto Matsukawa, Yusuke Yokota, Ryuta Urakawa, Susumu Tanaka

**Affiliations:** 1 Department of Oral and Maxillofacial Surgery, Graduate School of Dentistry, Osaka University, 1-8 Yamadaoka, Suita 565-0871, Japan; fujimoto.yukari.dent@osaka-u.ac.jp (Y.F.); matsukawa.makoto.dent@osaka-u.ac.jp (M.M.); yokota.yusuke.dent@osaka-u.ac.jp (Y.Y.); tanaka.susumu.dent@osaka-u.ac.jp (S.T.); 2 Unit of Dentistry, Osaka University Hospital, 2-15 Yamadaoka, Suita 565-0871, Japan; 3 Department of Pharmacy, Osaka University Dental Hospital, 1-8 Yamadaoka, Suita 565-0871, Japan; urakawa-r@office.osaka-u.ac.jp; 4 Department of Clinical Pharmacy Research and Education, Graduate School of Pharmaceutical Sciences, Osaka University, 1-6 Yamadaoka, Suita 565-0871, Japan

**Keywords:** postoperative pneumonia, postoperative infection, perioperative oral care

## Abstract

Numerous studies report that perioperative oral care decreases the frequency of postoperative pneumonia or infection. However, no studies have analyzed the specific impact of oral infection sources on the postoperative course, and the criteria for preoperative dental care differ among institutions. This study aimed to analyze the factors and dental conditions present in patients with postoperative pneumonia and infection. Our results suggest that general factors related to postoperative pneumonia, including thoracic surgery, sex (male > female), the presence or absence of perioperative oral management, smoking history, and operation time, were identified, but there were no dental-related risk factors associated with it. However, the only general factor related to postoperative infectious complications was operation time, and the only dental-related risk factor was periodontal pocket (4 mm or higher). These results suggest that oral management immediately before surgery is sufficient to prevent postoperative pneumonia, but that moderate periodontal disease must be eliminated to prevent postoperative infectious complication, which requires periodontal treatment not only immediately before surgery, but also on a daily basis.

## 1. Introduction

Long surgeries under general anesthesia are highly invasive and increase the risk of postoperative complications such as pneumonia or infection. In Japan, the incidence of postoperative pneumonia is reported to be 2.6–3.5%, and the incidence of postoperative infectious complication is reported to be 5.4% [1,2]. Several studies have examined the risk factors for postoperative pneumonia or infection from various views, and many of these studies report that perioperative oral care decreases the frequency of postoperative pneumonia or infection [1,3,4,5,6,7,8,9,10,11,12]. Besides perioperative oral care, removing infection sources from the oral cavity before surgery is recommended, and preoperative caries treatment and tooth extraction may be recommended in some cases, especially for patients undergoing chemotherapy [12,13,14,15]. Dental caries, periodontal disease, and dental infections are bacterial infections that often exist in the oral cavity in a state of chronic infection. Pathogenic microorganisms and inflammation-related substances, such as cytokines, can enter the blood from the foci of infection, and bacteremia has been reported to be caused by brushing teeth on a daily basis [16]. Additionally, the immunosuppressive state associated with surgical trauma has been reported to cause bacteremia, sepsis, and focal infection through the blood flow [17]. Several studies have analyzed the impact of oral infection sources on the postoperative course, and the importance of preoperative dental care has been increasingly recognized, but the criteria for preoperative dental care differ among institutions [13,14,18]. Many previous reports have focused on specific organs or limited their analysis to either pneumonia or infectious complications, but no reports have compared both in the same group.

In Japan, “perioperative oral management” was newly established as a medical fee in 2012 and has become widely used. The main objectives of perioperative oral management are to prevent and reduce adverse events, and to maintain and improve nutritional status and quality of life through comprehensive oral care, the control of dental infection foci, maintenance, and improvement of oral functions. Perioperative oral management has spread nationwide after the covering of fees by the national health insurance system, and has shown many benefits for both patients and medical staff. However, it is limited by disease and organ failure, and the scientific basis for its setting is ambiguous.

This study aimed to focus on both postoperative pneumonia and infectious complications and to conduct a retrospective study on the factors causing postoperative complications from the viewpoint of both general factors and dental-related risk factors.

## 2. Materials and Methods

### 2.1. Study Design and Population

A total of 10,628 patients who had undergone surgery under general anesthesia at the Osaka University Hospital between April 2016 and March 2017 were enrolled. Of the 10,628 patients, 2208 who had undergone operations lasting at least 3 h (scheduled surgery) and aged 20 years or older were included in this study. Cases of intravenous sedation, local anesthesia surgery, after-hours emergency surgery (surgery performed after evening, late at night, or outside normal hours, such as Sundays and holidays), and surgery performed on children under 20 years old were excluded. The medical records of the patients were retrospectively reviewed.

This study was independently approved by the Osaka University Graduate School of Medicine Ethics Committee (No. 17286). Clinical investigations were conducted in accordance with the principles of the Declaration of Helsinki.

### 2.2. Measures

Postoperative pneumonia and infectious complications were investigated in all patients. The predictor variables included patient factors—including age at surgery, gender, Eastern Cooperative Oncology Group Performance Status (PS) [19], body mass index (BMI), smoking habits, drinking habits, diabetes mellitus, severe heart disease (grade 3 or higher according to the New York Heart Association Classification [20]), and severe pulmonary disease (<60% of %vital capacity or <50% of %forced expiratory volume in one second)—and treatment factors—including the presence or absence of perioperative oral management, risk of general anesthesia (evaluated using the American Society of Anesthesiologists (ASA) physical status classification [21]), disease leading to surgery, the extent of surgical wounds, operation time, and amount of blood loss. PS aims to quantify general well-being and the activities of daily life of a patient. There are several scores obtained from PS, but the Eastern Cooperative Oncology Group score was used in this study [19]. A score of 0 is ‘Asymptomatic’, a score of 1 is ‘Symptomatic but completely ambulatory’, a score of 2 is ‘Symptomatic, <50% in bed during the day’, a score of 3 is ‘Symptomatic, >50% in bed, but not bedbound’, a score of 4 is ‘Bedbound’, and a score of 5 is ‘Death’.

Additionally, the details of the dental condition of 295 patients (13.4%) who had undergone perioperative oral management at our dental clinic were investigated. The relationship between postoperative pneumonia and infection was also examined in relation to the number of remaining teeth, periodontal pocket (4 mm or higher), the presence or absence of bleeding or pus discharge on probing, tooth mobility according to Miller classification [22], and preoperative tooth extraction, which were considered sources of infection.

### 2.3. Statistical Analysis

The risk factors for postoperative pneumonia and infection were examined using the chi-square test of independence, the Student’s *t*-test, and logistic regression. Statistical analysis was performed using JMP ver.13 (JMP Statistical Discovery LLC., Cary, NC, USA). *p*-values less than 0.05 were considered statistically significant.

## 3. Results

Of the 2208 patients included in this study, 1175 (53.2%) were males and 1033 (46.8%) were females (Table 1). The number of males (*n* = 197) who visited our dental clinic was higher than that of females (*n* = 134, *p* = 0.012). A significant difference in female age was observed between the groups with and without perioperative oral management; female patients with perioperative oral management had a mean age of 64.3 ± 14.6 years, whereas female patients without perioperative care had a mean age of 57.3 ± 13.8 years, with those with oral care being significantly older. Of the patients who visited our dental clinic, 191 patients were scheduled for cardiovascular surgery, including 79 patients (41.4%) scheduled for valve replacement, 33 (17.3%) for valve repair, 27 (14.1%) for coronary or aortic bypass surgery, 17 (8.9%) for arrhythmia surgery, 13 (6.8%) for aortic aneurysmectomy, 8 (4.2%) for implantable artificial heart replacement, and 14 (7.3%) for other treatments. The most common surgical site was the abdomen, with 64 patients, including 36 patients (56.3%) scheduled for esophageal malignant tumor resection (23 for gastrointestinal reconstruction surgery and 13 for laparoscopic surgery), 5 (7.8%) for pancreatic head tumor resection, 4 (6.3%) for gastrectomy, 3 (4.7%) for living-donor liver transplantation, and 16 (25.0%) for other treatments. Overall, there were more males than females, and older patients tended to attend referrals more than younger patients.

**Figure 1:**
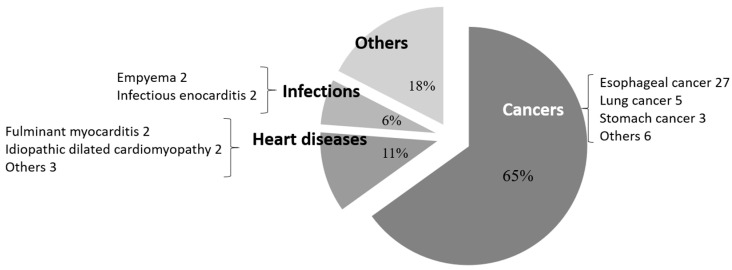
Classification of cases that caused postoperative pneumonia. Cases of malignant tumors, especially esophageal cancer, lung cancer, and stomach cancer, were most frequent, followed by cardiac disease and patients who originally underwent operations for the treatment of infection, such as empyema and infectious endocarditis. Postoperative pneumonia tended to occur mostly after thoracic surgery.

**Figure 2:**
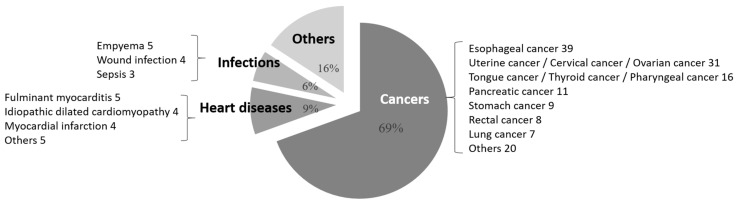
Classification of cases that caused postoperative infectious complications. These results suggest that postoperative infectious complications can occur at surgical sites other than those of thoracic surgery, compared to postoperative pneumonia.

Malignant tumors, especially esophageal cancer, were the most frequent cases of postoperative pneumonia and infectious complications. Thereafter, the most frequent cases of postoperative pneumonia were lung cancer, stomach cancer, and other malignant tumors, followed by cardiac disease (fulminant myocarditis, idiopathic dilated cardiomyopathy, and others), and those in which patients originally underwent operations for the treatment of infection (empyema and infectious endocarditis) (Figure 1). In other words, postoperative pneumonia occurred mostly after thoracic surgery. However, besides esophageal cancer, wound infections occurred frequently, such as uterine cancer in the female genital organs; tongue and larynx cancer in the mid-pharynx; pancreatic cancer in the digestive organs; and lung cancer in the respiratory organs. Meanwhile, non-malignant tumors were most common in those with cardiac disease and infections (pyothorax, wound infection, and sepsis), covering multiple organs besides the chest (Figure 2).

**Figure 3:**
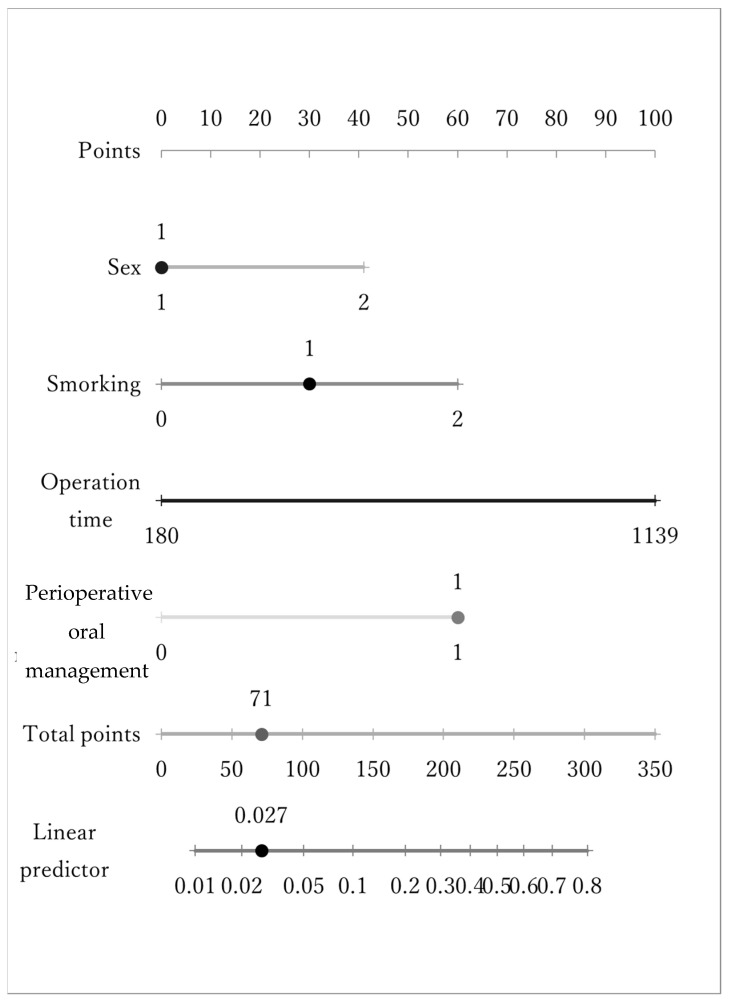
A nomogram of risk factors of postoperative pneumonia. For sex, 1 indicates female and 2 indicates male. For smoking, 0 indicates never, 1 indicates stop smoking before 1 year and 2 indicates continuing. For perioperative oral management, 0 indicates none and 1 indicates done.

Of the 2208 patients, 63 patients developed postoperative pneumonia, with an incidence rate of 2.85%. As for the risk of postoperative pneumonia, significant differences were observed in systemic risk in terms of sex, the presence or absence of perioperative oral management, PS, smoking history, anesthesia risk (ASA physical status classification), operation time, and amount of blood loss (Table 2). Thus, the incidence of postoperative pneumonia was significantly higher in males, those without perioperative oral management, those with a high class in the ASA physical status classification, those with long operation times, and those with heavy bleeding. However, there were no significant differences in age, BMI, the presence or absence of severe heart disease, the presence or absence of severe pulmonary disease, or surgical invasiveness. In the multivariate analysis, significant differences were observed in the presence or absence of perioperative oral management, smoking history, operation time, and amount of blood loss, and the nomogram derived from these results can enable prediction of the risk value of the onset of postoperative pneumonia (Table 3, Figure 3). However, no significant differences were observed in the oral risk factors, such as tooth number, EPP, tooth mobility, the presence or absence of bleeding of the pockets, the presence or absence of pus discharge from the pockets, or the presence or absence of extraction before operation (Table 4).

This nomogram, made from the results of the multivariate analysis of general risk factors for postoperative pneumonia, can enable prediction of the risk value of the onset of postoperative pneumonia. (For sex, 1 indicates female and 2 indicates male. For smoking, 0 indicates never smoking, 1 indicates the cessation of smoking before 1 year, and 2 indicates continuing smoking. For perioperative oral management, 0 indicates none and 1 indicates that it has been carried out.)

Regarding the risk of postoperative infectious complications, a significant difference was observed in the systemic factors, such as the presence or absence of perioperative oral management, PS, operation time, amount of blood loss, and the extent of incision (Table 5). Thus, the incidence of postoperative infectious complications was significantly higher in those without perioperative oral management, those with a high PS score, those with long operation times, and those with heavy bleeding. However, there were no significant differences in age, sex, BMI, the presence or absence of smoking history, the presence or absence of severe heart disease, the presence or absence of severe pulmonary disease, the severity of the ASA physical status classification, or surgical invasiveness. The multivariate analysis showed a significant difference only in the operation time (Table 6). As for oral risk factors, a significant difference was observed in those with periodontal pockets of ≥4 mm. However, no significant difference was observed in the presence or absence of bleeding or pus discharge, tooth mobility, or preoperative tooth extraction (Table 7).

## 4. Discussion

This study shows that common risk factors for both postoperative pneumonia and postoperative infectious complication were the absence of perioperative oral management, PS, operation time, and blood loss. Moreover, dental treatment and oral care just before surgery could sufficiently reduce the risk of postoperative pneumonia and infection. Additionally, we sometimes hesitate to extract teeth diagnosed to be the source of infection immediately before surgery because they may temporarily cause bacteremia, increasing the risk of postoperative pneumonia or infection. However, such cases were not reported. The cases in which tooth extraction just before surgery was unsuccessful were not due to infection, but in patients undergoing cardiovascular surgery, bleeding was difficult to stop. Several cases were reported in which bleeding was required to stop even on the morning of the operation.

Thoracic surgery, sex (male > female), the presence or absence of perioperative oral management, smoking history, and operation time were identified as factors related to postoperative pneumonia. Additionally, esophageal cancer was the most common cause of postoperative pneumonia, probably due to aspiration. Many previous reports have relied on site-specific or disease-specific statistics, and the authors have only empirically known in which areas postoperative pneumonia was more likely to occur. This study included all regions and found that postoperative pneumonia is more likely to occur after surgery in the thoracic region. Preoperative professional toothbrushing, tooth scaling, and tongue cleaning have been reported to prevent the onset of postoperative pneumonia and work to shorten the postoperative oral intake interruption period and postoperative hospital stays following gastrointestinal cancer surgery or lung cancer surgery [1,3,4,5,6,7,8,9,10,11]. However, there would be a similar tendency even if surgeries for other organ cancers or surgeries other than cancer surgeries were included in this study. These results suggest that perioperative oral management and tooth scaling or brushing reduce bacteria on the gingival margin, thus preventing postoperative pneumonia, according to a previous report [23].

The incidence of postoperative infectious complication was significantly higher with moderate periodontal disease. It is a severe judgment to say that it is not good to have a 4 mm or more periodontal pocket due to its risk, but this is consistent with previous reports [24,25]. For more than a decade, periodontal disease has been linked to a variety of systematic diseases, such as diabetes or heart disease [26]. Therefore, it is possible that periodontal bacteria are hematogenously transported to the wound after surgery, causing infection. To support this, however, periodontal bacteria would have to be detected in the infected wound, but this was not observed in this study, and in our experience, periodontal bacteria have never been detected in such cases. Recently, Arimatsu et al. reported that patients with periodontal disease are in an environment where periodontal disease-causing bacteria constantly flow into the digestive tract, which disrupts the balance of the intestinal flora and increases the amount of bacteria-derived toxins in the blood, leading to inflammation in other organs [27]. Furthermore, the results of this study suggest a relationship between periodontal disease-causing bacteria and postoperative infection after gastrointestinal surgery. Therefore, strengthening subgingival bacterial control, such as periodontal disease treatment before surgery, is thought to prevent postoperative infection. Additionally, oral care just before surgery is insufficient, and taking care of periodontal disease on a daily basis is essential, since even moderate periodontal disease may have an effect.

However, there was a combination of different surgeries in this study, so the reasons for the longer surgery times were varied. Moreover, operation time is not necessarily proportional to the degree of invasiveness. Damage from the amount of blood loss also differed between body sizes, so it may have been necessary to examine the progression of anemia. In addition, the presence or absence of blood transfusion was not examined. Since this is a retrospective study, selection bias may include the following. Since the study focuses only on surgeries at one hospital, the study is limited to the treatment methods of that department. The skill of the surgeon and postoperative management may not be the same as those of other hospitals. Because we are a university hospital, we handle many difficult cases and our caseload is biased. A multicenter study is needed to generalize the results.

From now on, educational activities will be conducted in the hospital to encourage people to attend family dentists and visit our dental clinic during hospitalization, especially for patients who are expected to have a long-lasting operation, gastrointestinal surgery, or surgery for malignant tumors.

## 5. Conclusions

The general factors related to postoperative pneumonia were thoracic surgery, sex (male > female), the presence or absence of perioperative oral management, smoking history, and operation time, but there were no dental-related risk factors associated with it. However, the only general factor related to postoperative infectious complications was operation time, and the only dental-related risk factor was EPP. These results suggest that oral management immediately before surgery is sufficient to prevent postoperative pneumonia, but that moderate periodontal disease must be eliminated to prevent postoperative infectious complication, which requires periodontal treatment not only immediately before surgery, but also on a daily basis.

## Figures and Tables

**Table 1:** Patients’ characteristics.

	With Perioperative Oral Management	Without Perioperative Oral Management	*p*-Value
Male (%) (*n* = 1175)	197 (16.8) *	978 (83.2)	
Age (y)	63.4 ± 14.3	63.1 ± 14.8	
Female (%) (*n* = 1033)	134 (13.0) *	899 (87.0)	
Age (y)	64.3 ± 14.6	57.3 ± 13.8	<0.001 ***
Total (%) (*n* = 2208)	331 (15.0)	1877 (85.0)	
Age (y)	63.8 ± 14.0	60.4 ± 15.5	
Surgical site			
Abdomen (%) (*n* = 631)	64 (10.1)	567 (89.9)	-
Heart (%) (*n* = 626)	191 (30.5)	435 (69.5)	-
Genitals (%) (*n* = 233)	4 (1.7)	229 (98.3)	-
Urinary tract and adrenal glands (%) (*n* = 205)	8 (3.9)	197 (96.1)	-
Ear, nose, and throat (%) (*n* = 223)	26 (11.7)	197 (88.3)	-
Thorax (%) (*n* = 157)	18 (11.5)	139 (88.5)	-
Neurocranium (%) (*n* = 138)	3 (2.2)	135 (97.8)	-
Breast (%) (*n* = 123)	1 (0.8)	122 (99.2)	-
Musculoskeletal system, limbs, and trunk (%) (*n* = 89)	4 (4.5)	85 (95.5)	-
Others (%) (*n* = 118)	3 (2.5)	115 (97.5)	-

* = 0.012 (*p* < 0.05); *** *p* < 0.001.

**Table 2:** Univariate analysis of general risk factors for postoperative pneumonia.

		With Pneumonia (*n* = 63)	Without Pneumonia (*n* = 2145)	*p*-Value
Age (y)	Mean ± SD	65.7 ± 11.9	60.7 ± 15.4	0.0628
Sex	Male (%)	51 (81.0)	1124 (52.4)	<0.001 ***
	Female (%)	12 (19.0)	1021 (47.6)	
Perioperative oral management	Yes (%)	23 (36.5)	308 (14.4)	<0.001 ***
	No (%)	40 (63.5)	1837 (85.6)	
PS	Score 0 (%)	13 (20.6)	1093 (51.0)	<0.001 ***
	Score 1 (%)	36 (57.1)	628 (29.3)	
	Score 2 (%)	6 (9.5)	252 (11.8)	
	Score 3 (%)	7 (11.1)	140 (6.5)	
	Score 4 (%)	1 (1.6)	32 (1.5)	
BMI	Mean ± SD	22.3 ± 3.6	23.4 ± 10.8	0.3836
Smoking	Never (%)	35 (55.6)	1730 (80.7)	<0.001 ***
	Stopped smoking before 1 year (%)	19 (30.2)	328 (15.3)	
	Continuing (%)	9 (14.3)	87 (4.1)	
Severe heart disease (NYHA ≧ 3)	Yes (%)	4 (6.3)	97 (4.5)	0.4941
	No (%)	59 (93.7)	2048 (95.5)	
Severe pulmonary disease (%VC < 60%, FEV1.0% < 50%)	Yes (%)	3 (4.8)	55 (2.6)	0.2829
	No (%)	60 (95.2)	2090 (97.4)	
ASA physical status classification	Class 1 (%)	2 (3.2)	601 (28.0)	<0.001 ***
	Class 2 (%)	46 (73.0)	1075 (50.1)	
	Class 3 (%)	12 (19.0)	433 (20.2)	
	Class 4 (%)	3 (4.8)	33 (1.5)	
	Class 5 (%)	0 (0.0)	2 (0.1)	
Operation time (h)	Mean ± SD	7.0 ± 2.8	5.5 ± 2.3	<0.001 ***
Blood loss (mL)	Mean ± SD	904.2 ± 2493.4	652.7 ± 1456.4	0.0032 **
Surgical invasiveness	Endoscopy (%)	26 (41.3)	918 (42.8)	0.9656
	Thoracotomy or laparotomy (%)	37 (58.7)	1221 (56.9)	
	Thoracotomy and laparotomy (%)	0 (0.0)	6 (0.3)	

** *p* < 0.005; *** *p* < 0.001. PS: performance status; BMI: body mass index; VC: vital capacity; FEV1.0: forced expiratory volume in one second; ASA: American Society of Anesthesiologists.

**Table 3:** Multivariate analysis of general risk factors for postoperative pneumonia.

	Odds Ratio	Lower 95% CI	Upper 95% CI	*p*-Value
Sex	2.7759	1.4279	5.3965	0.0026 **
Perioperative oral management	2.6911	1.5242	4.7513	<0.001 ***
Performance status	1.2281	0.9462	1.5940	0.1403
Smoking	2.0467	1.4137	2.9627	<0.001 ***
ASA physical status	1.2374	0.8282	1.8488	0.1746
Operation time	1.0684	1.0210	1.1180	0.0033 **
Blood loss	0.3369	4.7060	4054.4	0.4118

** *p* < 0.005; *** *p* < 0.001. CI: confidence interval; ASA: American Society of Anesthesiologists.

**Table 4:** Univariate analysis of dental-related risk factors of postoperative pneumonia.

		With Infection (*n* = 18) (Excluded 4 Edentulous Patients)	Without Infection (*n* = 277) (Excluded 2 Edentulous Patients)	*p*-Value
Tooth number	Mean ± SD	20.0 ± 8.7	21.8 ± 7.8	0.3391
EPP (>4 mm)	Yes (%)	14 (77.8)	206 (74.4)	0.7475
	No (%)	4 (22.2)	71 (25.6)	
More than 1 tooth with mobility 3	Yes (%)	7 (38.9)	76 (27.4)	0.2951
	No (%)	11 (61.1)	201 (72.6)	
Bleeding of pocket	Yes (%)	15 (83.3)	190 (68.6)	0.1881
	No (%)	3 (16.7)	87 (31.4)	
Pus discharge from pocket	Yes (%)	1 (5.5)	3 (1.1)	0.1119
	No (%)	17 (94.4)	274 (98.9)	
Extraction before operation	Yes (%)	4 (22.2)	84 (30.3)	0.4665
	No (%)	14 (77.8)	193 (69.7)	

SD: standard deviation, EPP: examination of periodontal pocket.

**Table 5:** Univariate analysis of general risk factors for postoperative infectious complications.

		With Infection (*n* = 203)	Without Infection (*n* = 2005)	*p*-Value
Age (y)	Mean ± SD	60.6 ± 15.3	60.9 ± 15.4	0.7781
Sex	Male (%)	105 (51.7)	1070 (53.4)	0.6549
	Female (%)	98 (48.3)	935 (46.6)	
Perioperative oral management	Yes (%)	45 (22.2)	286 (14.3)	0.0026 **
	No (%)	158 (77.8)	1719 (85.7)	
Performance status	Score 0 (%)	87 (42.9)	1058 (52.8)	0.0361 *
	Score 1 (%)	76 (37.4)	558 (27.8)	
	Score 2 (%)	13 (6.4)	245 (12.2)	
	Score 3 (%)	21 (10.3)	126 (6.3)	
	Score 4 (%)	6 (3.0)	27 (1.3)	
BMI	Mean ± SD	23.4 ± 11.1	22.5 ± 4.42	0.2186
Smoking	Never (%)	160 (78.8)	1605 (80.0)	0.7980
	Stopped smoking before 1 year (%)	35 (17.2)	312 (15.6)	
	Continuing (%)	8 (3.9)	88 (4.4)	
Severe heart disease (NYHA ≥ 3)	Yes (%)	15 (7.3)	86 (42.9)	0.4941
	No (%)	188 (92.6)	1919 (95.7)	
Severe pulmonary disease (%VC < 60%, FEV1.0% < 50%)	Yes (%)	9 (4.4)	49 (24.4)	0.0912
	No (%)	194 (95.6)	1956 (97.6)	
ASA physical status classification	Class 1 (%)	43 (21.2)	561 (28.0)	0.0962
	Class 2 (%)	113 (55.7)	1008 (50.3)	
	Class 3 (%)	37 (18.2)	408 (20.3)	
	Class 4 (%)	10 (4.9)	26 (1.3)	
	Class 5 (%)	0 (0.0)	2 (0.1)	
Operation time (h)	Mean ± SD	6.6 ± 2.9	5.4 ± 2.2	<0.001 ***
Blood loss (mL)	Mean ± SD	907.6 ± 1878.1	637.8 ± 1455.3	0.0032 **
Surgical invasiveness	Endoscopy (%)	66 (32.5)	878 (43.8)	0.0072 **
	Thoracotomy or laparotomy (%)	136 (67.0)	1122 (56.0)	
	Thoracotomy and laparotomy (%)	1 (0.5)	5 (0.3)	

* *p* < 0.05, ** *p* < 0.005, and *** *p* < 0.001. SD: standard deviation.

**Table 6:** Multivariate analysis of general risk factors for postoperative infectious complications.

	Odds Ratio	Lower 95% CI	Upper 95% CI	*p*-Value
Perioperative oral management	1.4255	0.9800	2.0746	0.0637
Performance status	1.0810	0.9304	1.2559	0.3699
Operation time	1.1177	1.0648	1.1732	<0.001 ***
Blood loss	0.9999	0.9999	1.0000	0.4116
Surgical invasiveness	1.2794	0.9189	1.7814	0.1761

*** *p* < 0.001. CI: confidence interval.

**Table 7:** Univariate analysis of dental-related risk factors of postoperative infectious complications.

		With Infection (*n* = 36) (Excluded 4 Edentulous Patients)	Without Infection (*n* = 259) (Excluded 2 Edentulous Patients)	*p*-Value
Tooth number	Mean ± SD	20.8 ± 9.3	21.9 ± 7.7	0.4294
EPP (>4 mm)	Yes (%)	22 (61.1)	198 (76.4)	0.0477 *
	No (%)	14 (38.9)	61 (23.6)	
More than 1 tooth with mobility 3	Yes (%)	11 (30.1)	72 (27.8)	0.7304
	No (%)	25 (69.4)	187 (72.2)	
Bleeding of pocket	Yes (%)	24 (66.7)	181 (70.0)	0.6447
	No (%)	12 (33.3)	76 (29.3)	
Pus discharge from pocket	Yes (%)	1 (2.8)	4 (1.5)	0.0976
	No (%)	35 (97.2)	25 (69.4)	
Extraction before operation	Yes (%)	12 (33.3)	76 (29.3)	0.6240
	No (%)	24 (66.7)	183 (70.7)	

* *p* < 0.05, EPP: examination of periodontal pocket.

## Data Availability

All the materials and information are available upon request via e-mail to the corresponding author.
